# Maternal *Lactobacillus johnsonii* Supplementation Is Associated with Altered Offspring Cecal Microbiome and Reduced Cockroach Allergen-Induced Airway Responses in Mice

**DOI:** 10.3390/biomedicines14091892

**Published:** 2026-08-25

**Authors:** Llilian Arzola Martínez, Alexander D. Ethridge, Andrew J. Rasky, Susan Morris, Kazuma Yagi, Catherine Ptaschinski, Wendy Fonseca, Nicholas W. Lukacs

**Affiliations:** 1 Department of Pathology, University of Michigan, Ann Arbor, MI 48109, USA; larzolam@med.umich.edu (L.A.M.);; 2 Mary H. Weiser Food Allergy Center, University of Michigan, Ann Arbor, MI 48109, USA

**Keywords:** microbiome, allergy, probiotic

## Abstract

**Background**: Early-life microbial exposures shape immune maturation and can influence later susceptibility to allergic airway disease. While probiotics can modulate host immunity, determining whether maternal probiotic supplementation alone promotes changes in offspring microbiomes and the immune functions that reduce allergic disease remains incompletely defined. **Methods**: Female mice were gavaged with *Lactobacillus johnsonii* (Lj) daily for 7 days prior to mating, and then twice weekly until delivery. Offspring were sensitized and challenged with cockroach allergen (CRA), beginning at 5 weeks of age, for 3 weeks to initiate asthmatic-type responses. Airway physiology (methacholine-induced airway hyperreactivity; AHR), lung-mucus-related gene expression (*Muc5ac*, *Gob5*), and lymph node restimulation responses were assessed. **Results**: Offspring from Lj-supplemented dams exhibited reduced AHR and decreased induction of Muc5ac and Gob5 following the final CRA challenge, accompanied by diminished Th cell cytokine production upon lymph node restimulation. Maternal Lj supplementation produced a significant shift in offspring cecal microbiome structure at 5 weeks, including altered phylum-level composition and a discrete set of significantly changed OTUs, including Akkermansia. PICRUSt2 analyses predicted coordinated differences in microbial metabolic potential across multiple pathways, consistent with functional reprogramming of the gut microbiome. Bone marrow dendritic cells (BMDC) derived from adult offspring of Lj-supplemented dams showed attenuated inflammatory programming after RSV or TLR7 stimulation, with reduced expression of innate immune cytokines compared to PBS-supplemented dams. **Conclusions**: The relatively long-term effects were associated with reduced allergic airway disease severity, supporting maternal probiotic supplementation as a potential strategy to lower offsprings’ risk of allergic airway pathology.

## 1. Introduction

Early life represents a uniquely sensitive window, during which the developing immune system is established to distinguish innocuous environmental antigens from true threats. During this period, the infant microbiome rapidly assembles across mucosal sites, especially the gut, providing significant antigenic exposure and immunoregulatory signals that shape barrier integrity and immune education thought to regulate overall inflammation [1,2,3,4,5,6,7,8]. A growing body of epidemiologic and experimental evidence supports the concept that perturbations to this early microbial community can imprint durable immune phenotypes, thereby altering susceptibility to allergic disease long after infancy. Clinical birth cohorts reinforce that environment and lifestyle are major determinants of early-life microbiome assembly, with factors such as urbanization versus farm/rural living, diet, delivery mode, and antibiotic exposure producing reproducible shifts in infant microbial trajectories [9,10,11,12,13,14,15,16,17,18,19]. Importantly, these cohort studies show that early dysbiosis can precede and predict later atopy and asthma, supporting a model in which altered microbial inputs during critical developmental windows durably reprogram mucosal immune regulation.

Microbial colonization begins at birth and undergoes dynamic restructuring in the first months of life, coincident with the maturation of epithelial barriers and expansion of innate and adaptive immune networks [20,21]. Commensal bacteria influence these processes through multiple interlocking mechanisms, promoting mucus and tight-junction development, tuning innate sensing pathways, and driving tolerogenic antigen presentation which supports regulatory T-cell (Treg) differentiation and balanced type 1/type 2 immunity [22,23,24,25,26,27,28,29]. Microbiota-derived metabolites, particularly short-chain fatty acids and other small molecules generated from diet/microbe interactions, can further regulate immune and epithelial programs through epigenetic and receptor-mediated pathways, thereby shaping mucosal immune responses [30,31,32]. In parallel, the microbiome and the lung/gut axis provide bidirectional communication, through which microbial exposures in one compartment can influence immune reactions in another, offering a mechanistic basis for how early intestinal dysbiosis may affect later airway inflammation [33,34,35,36].

Consistent with these principles, early-life disruptions to the microbiome have been repeatedly associated with increased risk of allergic outcomes, including eczema, food allergy, allergic rhinitis, and asthma. Antibiotic exposure, altered diet and feeding practices, cesarean delivery, reduced microbial diversity, and loss of specific immunoregulatory taxa have each been linked to heightened allergic sensitization and exaggerated type 2 inflammation [33,37,38,39,40,41,42,43]. Importantly, these associations align with experimental studies demonstrating that defined microbial communities or probiotic interventions during pregnancy and infancy can modulate offspring immune development and reduce allergic airway disease in later life [44,45,46,47]. Together, these observations suggest that microbial exposures do not merely correlate with allergic disease, but can actively participate in programming long-term mucosal immunity.

Despite rapid progress, key gaps remain in defining which microbial features are causally responsible, when they must be present to confer protection, and how they mechanistically shape immune development across organs. In these studies, we utilized an established maternal probiotic supplementation model with Lj [47] to examine long-term protection from allergic asthma responses in offspring, including alterations in innate immune cells, particularly dendritic cells.

## 2. Materials and Methods

### 2.1. Lactobacillus johnsonii Preparation for Supplementation

*Lactobacillus johnsonii* was propagated from a glycerol stock using a selective Lactobacillus de Man, Rogosa and Sharpe (MRS) broth following a previously described protocol [47,48,49]. Briefly, Lj-inoculated MRS broth was cultured overnight at 37 °C until cells reached a stationary phase (Od_600_ = 0.89), and then centrifuged at 4000× *g* for 15 min at 4 °C. The cell pellet was resuspended in a 50:50 solution of MRS:50% glycerol (in saline). Aliquots of 500 µL were stored at −80 °C until used in murine studies. Serial dilutions were plated on MRS agar plates to determine viability counts of Lj batches. For murine studies, tubes were defrosted and centrifuged at 14,000 r.p.m at 4 °C and washed three times in sterile saline solution to remove glycerol. Cells were resuspended to 10^9^ CFU/mL in sterile saline solution and each mouse received 10^8^ CFU/mL in 100 µL of resuspended Lj. Viable Lj cell counts were assessed by plating the remaining suspension.

### 2.2. Studies Design

All experiments were approved by the Institutional Animal Care and Use Committee at the University of Michigan. All mice were maintained under pathogen-free conditions and purchased from The Jackson Laboratory for breeding.

### 2.3. Maternal Supplementation and CRA Model

BALB/c female mice, 7–8 weeks old, were gavaged with Lj or control saline daily for seven days before being paired with male mice for mating. Breeding was set up with 4 females in each of the PBS- and Lj-supplemented groups. Males were removed from the cage after 7 days and Lj supplementation continued twice weekly until delivery. Investigators were not formally blinded to maternal treatment during experimental procedures or outcome analysis. The mice were kept in standard cages, in company with their littermates of both sex and their mother. After weaning at 3 weeks, males and females were separated by sex (i.e., one cage for each sex) and mice were housed in the same room. Further, mice were not separated by their dams. Five weeks after birth, offspring were intratracheally sensitized with 200 PNU of CRA (Hollister Stier Allergy, Spokane, WA, USA) in 40 µL for three consecutive days (days 0, 1 and 2). On days 14, 20, 22 and 24, mice were locally challenged with 200 PNU of CRA in 40 µL of saline by the intratracheal route and mice were euthanized on day 25, 1 day after the final allergen challenge at the end of the model.

### 2.4. Airway Hyperresponsiveness (AHR)

Airway hyperreactivity was measured using a mouse plethysmograph designed for low tidal volumes (Data Sciences International, St. Paul, MN, USA) as a direct measure of airway resistance changes. Mice were anesthetized with sodium pentobarbital, and the trachea was cannulated with an 18-gauge metal tube. Animals were mechanically ventilated with a tidal volume of 200 µL at 120 breaths/min. Airway resistance was determined in the closed plethysmograph by measuring tracheal pressure relative to corresponding changes in chamber pressure, with values converted to resistance using computer-assisted calculations. After baseline resistance had stabilized, methacholine was administered by intravenous tail-vein injection at 0.350 mg/kg. The response was continuously monitored, and peak airway resistance following methacholine administration was recorded as the measure of airway hyperreactivity.

### 2.5. Lung Draining Lymph Node Restimulation and Cytokine Production Assay

Lung draining lymph nodes (LDLNs) were enzymatically digested by incubation with 1 mg/mL of collagenase A (Roche, Mannheim, Germany) and 20 U/mL DNAse I (Sigma-Aldrich, St. Louis, MO, USA) in RPMI 1640 with 10% FCS for 30 min with agitation at 37 °C. An 18-gauge needle (1 mL syringe) was used to further disperse the tissue. After red blood cell lysis, samples were passed through a 40 µm strainer and counted with an automated bright field cell counter (CellDrop, DeNovix, Wilmington, DE, USA). In total, 5 × 10^5^ cells from the suspension of LDLN were cultured in 96-well plates and restimulated with CRA for 48 h. Levels of T-cell-derived cytokines in supernatants were determined with a Bio-Plex assay (Bio-Rad Laboratories, Hercules, CA, USA).

### 2.6. RNA Purification and Quantitative RT-PCR

RNA was isolated from lung tissue and cell culture using TRIzol reagent according to the manufacturer’s instructions (Invitrogen, Waltham, MA, USA). cDNA was synthesized from 5 µg of RNA per sample using murine leukemia virus reverse transcriptase (MLV, Applied Biosystems, Foster City, CA, USA) by incubating the mix at 37 °C for 1 h, followed by incubation at 95 °C for 5 min to quench the reaction. Relevant gene expression was determined using predeveloped TaqMan Gene Expression assay probes/primers or custom primers to measure Muc5ac and Gob5 mRNA levels, as previously described [47]. Gene expression was normalized to *18S* rRNA gene as an internal control, and all reactions were run on the 7500 Real-Time PCR System (Applied Biosystems, Foster City, CA, USA). The 2^−∆∆Ct^ cycle threshold method was used to quantify fold change and 2^−∆Ct^ was used to calculate relative gene expression.

### 2.7. Lung Histology

A 4% (vol/vol) paraformaldehyde in saline (Sigma-Aldrich, St. Louis, MO) was used to perfuse lung lobes for preservation. Fixed lungs were embedded in paraffin and cut into five-micrometer sections. Lung sections were stained with periodic acid-Schiff (PAS) to detect mucus production, and with hematoxylin–eosin (H&E) to reveal inflammatory infiltrates. Photomicrographs were captured using a Zeiss Axio imager Z1 and AxioVision 4.8 software (Zeiss, Jena, Germany).

### 2.8. Bone Marrow Dendritic Cells (BMDC) Culture and Stimulation

BMDC were grown in complete RPMI 1640 medium containing 10 ng/mL of granulocyte macrophages colony-stimulating factor (GM-CSF, R&D Systems, Minneapolis, MN, USA). Cells were fed on days 3 and 5 with fresh GM-CSF. On day 6, when approximately 80% of the cells were CD11c+CD11b+, BMDC were seeded in 24-well tissue culture dishes and stimulated for 24 h with RSV (MOI of 1.0) or with the TLR7 agonist (Imiquimod-0.5 mg/mL) for 4 h. Cells were collected for RNA isolation (see Section 2.6).

### 2.9. Microbiome Analysis

The bacterial communities of the cecum prior to allergen sensitization were profiled using 16S rRNA amplicon sequencing. Whole ceca with contents from the offspring of non-supplemented and Lj-supplemented mothers were collected and snap frozen prior to allergen sensitization. Ceca were homogenized in PowerBead Tubes (Qiagen, Redwood City, CA, USA) and genomic DNA was isolated using the DNeasy Blood and Tissue Kit with a modified protocol that was described previously [50]. The V4 region of the 16S rRNA gene was amplified and sequenced, as previously described by the University of Michigan Microbiome Core [51]. Sequence data were processed and analyzed using mothur (v. 1.42.3) as previously described [52]. An isolation control receiving all steps of processing, including homogenization and DNA isolation, was included to identify potential contamination, which was removed using decontam with default parameters and the prevalence method [53]. The data were analyzed with mice separated into different cages based upon maternal treatment with cage effects considered as a variable. Significant operational taxonomic units (OTUs) were identified using mvabund (v. 4.2.1). Diversity metrics and community composition analyses were performed using vegan (v. 2.6-4.). Dam effects were not factored into the statistical analysis of the microbiome, as the offspring from each treatment, PBS or Lj supplementation, were from a single dam. Cages were separated by sex at weaning and tested for sex effect, which may also be considered cage effect due to separation.

### 2.10. PICRUSt2 Metagenomic Inference Analysis

All OTUs with relative abundance > 0.1% were included in PICRUSt2 analysis [54]. A .biom file was generated using count data corresponding to all OTUs that met the filtering criteria with the biomformat (v.1.20.0) package. Representative 16S rRNA OTU sequences exported from mothur were de-gapped and pruned to retain only filtered OTUs. Functional profiles were inferred with PICRUSt2 (v2.5.0) following the official tutorial and using default parameters. Predicted pathway abundances were normalized within each sample to total inferred functional abundance to generate relative abundances. Predicted pathways were consolidated into broad ‘Super Pathways’ using pathway ontology provided by MetaCyc database (v 26.5, accessed 22 February 2023) [55]. Cages were separated by sex at weaning.

### 2.11. Statistical Analysis

Group sizes were based on litter availability and sample sizes used in previously published experiments employing these models. No formal a priori power calculation was performed and formal randomization procedures were not used. Our studies could not assess sex differences as it was under-powered to make that determination. Overall, the study should therefore be considered exploratory.

### 2.12. Non-Microbiome Analysis

Data were analyzed using Prism v10 (GraphPad Software Inc., GraphPad Holdings LLC, San Diego, CA, USA). Data are presented as mean values ± SEM. Prior to parametric testing, data distributions were evaluated for normality using the Shapiro–Wilk test. Comparisons between two groups were performed using an unpaired, two-tailed *t*-test. Comparisons among three or more groups were analyzed by one-way ANOVA with Tukey’s multiple-comparison post hoc test. When data did not meet the assumptions for parametric testing, appropriate nonparametric tests were used. A *p* value < 0.05 was considered statistically significant.

### 2.13. Microbiome Analysis

Overall community composition was compared by principal component analysis using Euclidean distances of Hellinger-transformed count data followed by PERMANOVA with 1000 permutations. Phylum abundance was compared using Welch’s *t*-test with multiple hypothesis correction using Benjamini–Hochberg false discovery rate (FDR). Significantly altered OTUs were identified using the mvabund implementation of generalized linear models followed by ANOVA. Alpha-diversity (Shannon) and beta-diversity (Bray–Curtis) metrics were analyzed using Welch’s *t*-test. Importantly, the statistics were within group analysis of offspring from a single dam.

## 3. Results

**Figure 1:**
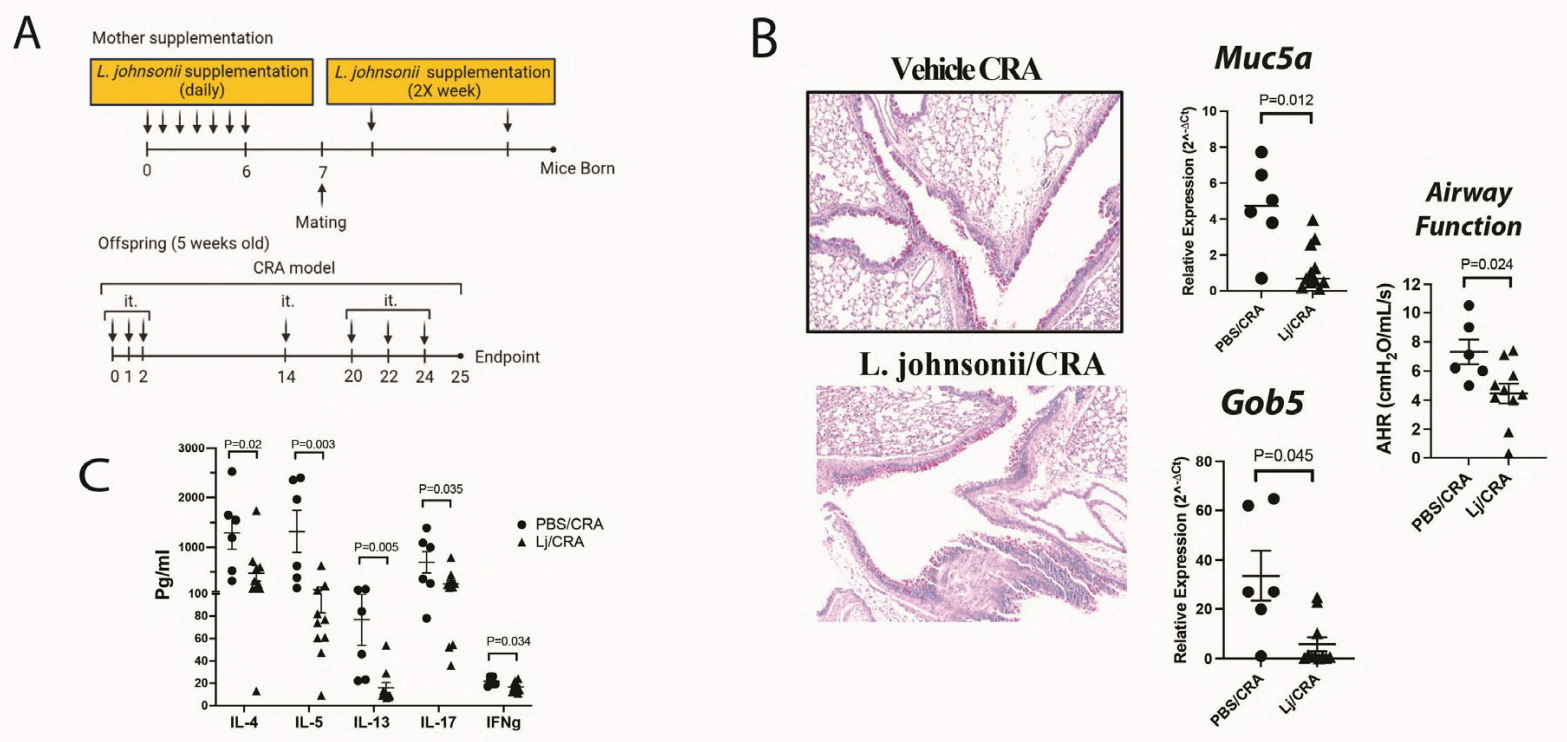
Maternal supplementation with *Lactobacillus jonhsonii* attenuates the severity of allergen-induced lung disease in offspring later in life. Female Balb/c mice were supplemented with Lj daily for seven days before mating, then twice a week during pregnancy. Five weeks after birth, offspring were given an allergen (cockroach allergen; CRA) sensitization model (**A**). In these offspringm PAS staining of histological section from lung showed less mucus production (red stain), as well as reduced expression of mucus-associated genes *Muc5ac* and *Gob5* (**B**). Offspring from Lj-supplemented dams also demonstrated reduced methacholine-induced airway hyperreactivity (AHR) compared to unsupplemented (**B**). Allergen-restimulated lung draining lymph nodes produced significantly reduced cytokines from offspring of Lj-supplemented mothers (**C**). Data represents the mean ± SEM from 6 mice in vehicle (from 2 dams) and 10 mice (from 3 dams) in Lj-supplemented dams.

**Maternal *Lj* supplementation reduced pathological allergic response in offspring.** It was previously determined that Lj supplementation in adult mice provides airway protection against allergen-induced exacerbated immune responses [49] and attenuates respiratory syncytial viral (RSV) infection [48,49]. In addition, our group previously showed that prenatal and postnatal exposure to Lj reduced the inappropriate Th2 immunity and mucogenic responses to RSV, respectively [47,48]. To determine whether maternal intervention with Lj reduces the risk of an allergic response in offspring later in life, female mice were gavaged daily with Lj for seven days prior to mating and twice weekly until delivery, as previously described [47]. Offspring were sensitized and challenged with CRA 5 weeks after birth as is described in the Methods (Figure 1A). Offspring from Lj-supplemented mothers showed a reduced airway hyperresponsiveness (AHR) to methacholine (Figure 1B) and the lung tissue exhibited decreased levels of mucus-related genes *Muc5ac* and *Gob5* (Figure 1B). Histopathologic examination showed modest differences in mucus production and inflammatory infiltration between offspring coming from non-supplemented and Lj-supplemented mothers (Figure 1B). However, cytokine production was diminished in offspring coming from Lj-supplemented mothers after in vitro restimulation of lung draining lymph nodes with CRA (Figure 1C). Thus, the outcome of maternal supplementation with Lj as a probiotic only in the mother prior to birth had a long-term effect on the offspring responses.

**Figure 2:**
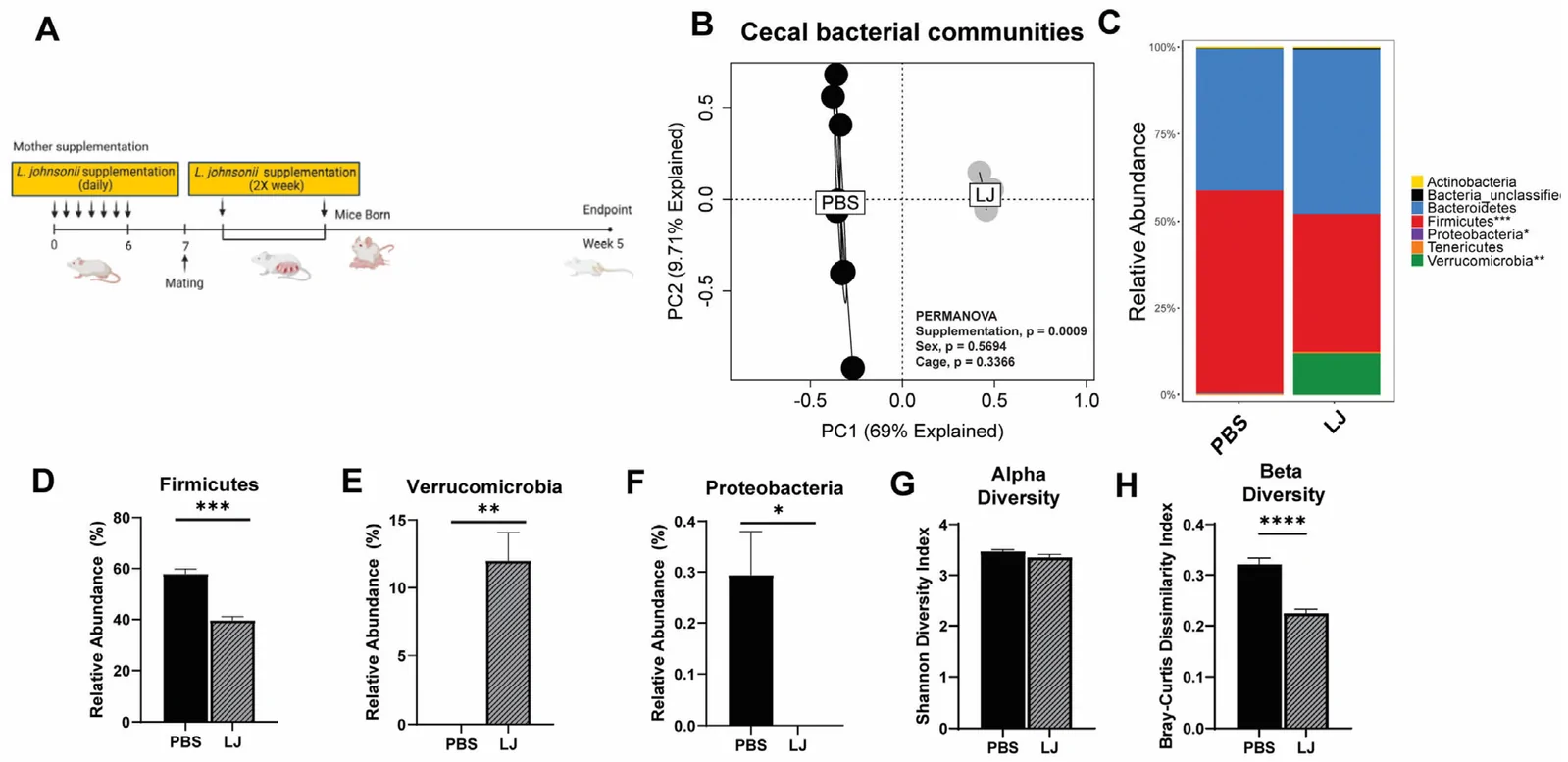
Maternal supplementation with Lj led to long-term gut microbiome alteration. The bacterial microbiome composition in the ceca of offspring from Lj-supplemented and PBS-supplemented mothers was examined prior to allergen sensitization at 5 weeks of age (**A**). Maternal supplementation led to the development of an altered gastrointestinal microbiome as shown in principal component analysis (**B**). The composition of the cecal microbiome in offspring shifted with Lj maternal supplementation (**C**). Abundance of Firmicutes was decreased with maternal Lj supplementation (**D**). Abundance of Verrucomicrobia was increased with maternal Lj supplementation (**E**). Abundance of Proteobacteria was decreased with maternal Lj supplementation (**F**). Alpha- and beta-diversity differences in the bacterial microbiome composition in the ceca of offspring from Lj-supplemented and PBS-supplemented mothers showed that alpha-diversity did not change, whereas offspring from Lj-supplemented mothers showed a lower beta-diversity (**G**,**H**). Data represents the mean ± SEM from 8 mice in control (4 female and 4 male) and 6 mice in supplemented (3 female and 3 male). **** *p* ≤ 0.0001, *** *p* ≤ 0.005, ** *p* ≤ 0.01, * *p* ≤ 0.05. Offspring in the supplemented mice were derived from a single dam.

**Maternal *Lj* supplementation modifies the gastrointestinal cecal microbiome into adulthood.** Maternal Lj supplementation produced a durable shift in offspring cecal microbial community structure that was still evident at the adult time point analyzed (week 5). In a separate cohort from those used in Figure 1, dams received Lj beginning 1 week prior to mating (daily), followed by a maintenance regimen during gestation (twice weekly) through delivery. Offspring in this analysis were derived from a single dam and were analyzed at 5 weeks of age (Figure 2A). Consistent with long-term microbiome “imprinting,” unsensitized 5-week-old offspring born to the Lj-supplemented dam displayed a clear separation in overall cecal bacterial community composition compared with offspring from unsupplemented dams, with the two groups clustering distinctly along the primary principal coordinate (PC1) axis (Figure 2B). At the phylum level, maternal Lj supplementation was associated with a compositional shift characterized by a reduction in the relative abundance of Firmicutes and Proteobacteria, along with a notable enrichment of Verrucomicrobia in the Lj group that was minimal/absent in controls (Figure 2C–F). While there was no difference in the Shannon diversity index (alpha-diversity), there was a significant reduction in the Bray–Curtis diversity index (beta-diversity) (Figure 2G,H), suggesting a more stable community, as previously described [47]. Together, these data suggest that maternal Lj supplementation produces a persistent, group-defining alteration in the offspring cecal microbiome that is maintained into early adulthood, that is a stable community structure prior to subsequent cockroach allergen sensitization and challenge.

**Figure 3:**
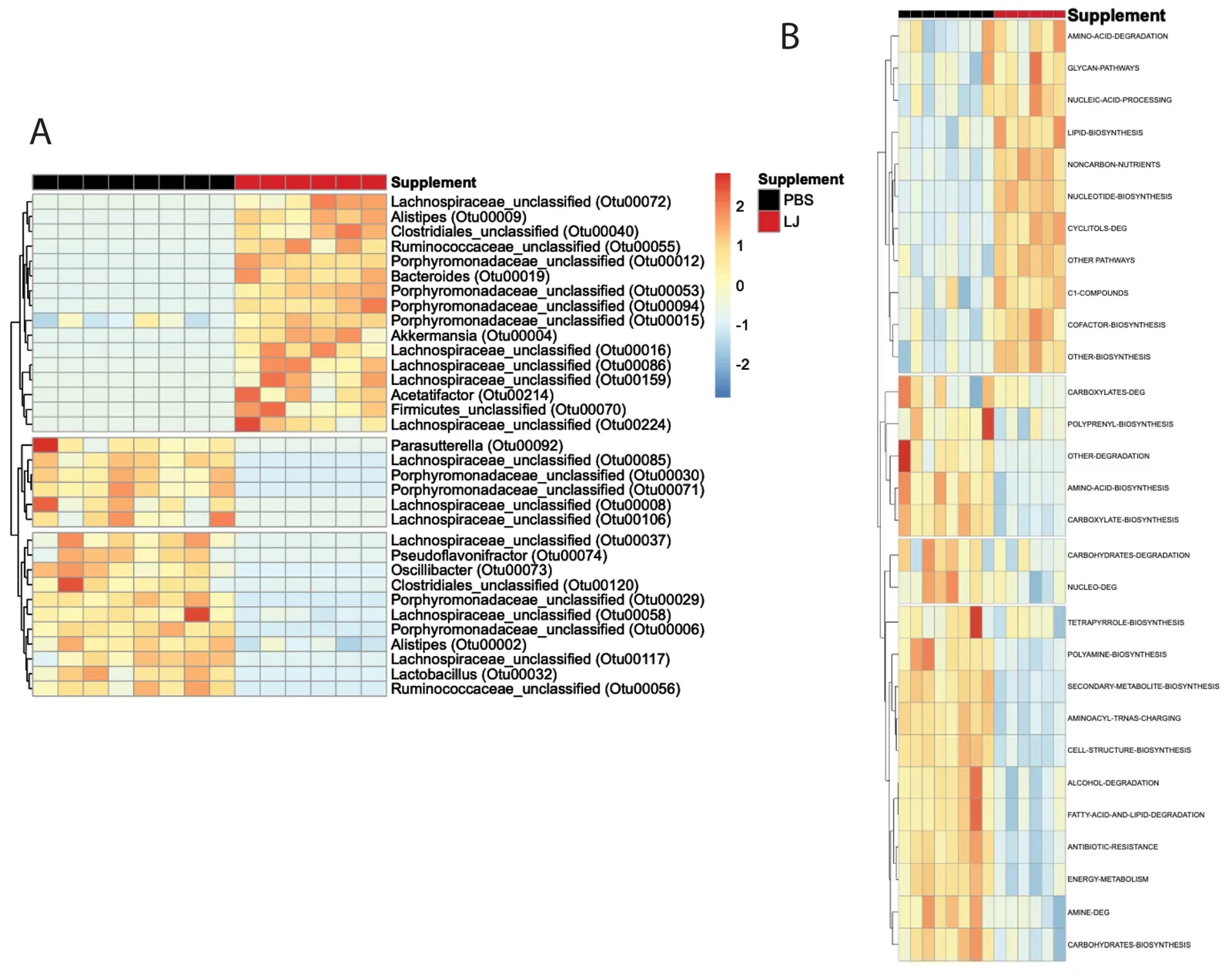
Maternal supplementation with Lj alters specific OTU as assessed by 16S sequencing and predicted (inferred) changes in metabolic pathway production due to the changed microbiome. Differences in the bacterial microbiome composition in the ceca of offspring from Lj-supplemented and PBS-supplemented mothers were further explored to highlight significantly altered OTUs (**A**). Those belonging to *Lachnospiraceae*, *Porphyromonadaceae*, and *Ruminococcaceae* families are altered due to maternal supplementation with Lj. The abundance of significantly altered MetaCyc-defined bacterial metabolic pathways was determined using PICRUSt2 and LEfSe. Relative abundances are scaled for each pathway to show fold change and hierarchically clustered (**B**). Data represents mean ± SEM from 8 mice in control (4 female and 4 male) and 6 mice in supplemented (3 female and 3 male). Offspring in the analysis were derived from a single dam.

At 5 weeks of age, maternal Lj supplementation was associated with a number of differentially abundant OTUs that segregated offspring by treatment group (Figure 3A). Hierarchical clustering of the significant OTUs identified two broad modules with an Lj-enriched cluster dominated by multiple Lachnospiraceae, Porphyromonadaceae, and additional taxa including Alistipes, Bacteroides, Akkermansia, Ruminococcaceae, and Acetatifactor, indicating expansion of several anaerobic commensal bacteria in offspring born to supplemented mothers. In contrast, a second module showed that the community was relatively depleted of numerous taxa such as Parasutterella, Pseudoflavonifractor, Oscillibacter, Clostridiales, along with Porphyromonadaceae and Lachnospiraceae members, consistent with targeted remodeling rather than a uniform increase across taxa (Figure 3A).

To infer the functional consequences of these compositional shifts, PICRUSt2 metagenomic prediction analyses illustrated marked differences in potential microbial metabolic capacity between groups (Figure 3B). Relative to controls, Lj offspring showed decreased pathways linked to fatty acid/lipid degradation, secondary metabolite biosynthesis, energy metabolism, antibiotic resistance, and multiple biosynthetic/degradation pathways including amino-acid biosynthesis, carbohydrate biosynthesis/degradation, polyamine and polyprenyl biosynthesis, and tetrapyrrole biosynthesis. Conversely, maternal Lj supplementation increased predicted pathways associated with glycan metabolism, amino-acid degradation, nucleic-acid processing and nucleotide biosynthesis, lipid biosynthesis, cofactor biosynthesis, and additional modules related to C1 compounds, noncarbon nutrients, cyclitols degradation, and other biosynthetic functions (Figure 3B). Together, these data indicate that maternal Lj supplementation is consistent with a durable microbiome community remodeling that is accompanied by a coordinated shift in predicted microbial metabolic programming in the offspring at 5 weeks of age.

**Maternal *Lj* supplementation altered bone marrow-derived dendritic cell production of innate cytokines**.

**Figure 4:**
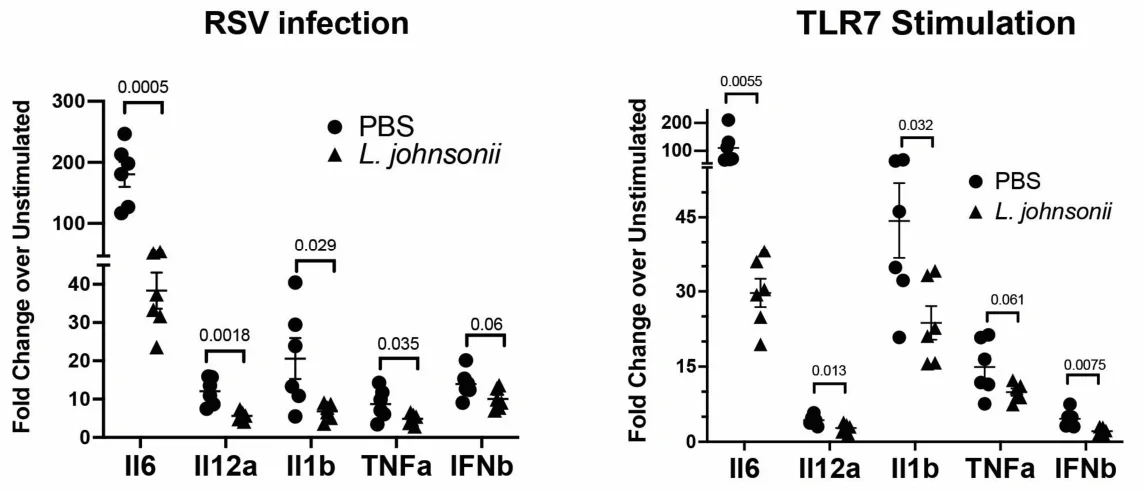
Innate immune cytokine expression is modified in bone marrow-derived DC (BMDC) from 5-week-old animals from offspring of Lj-supplemented dams. The regulatory role of maternal Lj supplementation in functional response of BMDC from offspring at 5 weeks of age was indicated by in vitro RSV infection (**left**) or TLR7 (**right**) stimulation reducing the expression of proinflammatory innate cytokines *Il6*, *Il12a*, *Il1b*, *TNFa*, and/or *IFNb*. Data represents the mean ± SEM from 6 mice in control (3 female and 3 male) and 6 mice in supplemented (3 female and 3 male). Offspring from the analysis were derived from a single dam.

In the same maternal Lj supplementation model, we also investigated the functional response of bone marrow-derived dendritic cells (BMDC). Our previous work showed that early-life events can influence immune cells, especially dendritic cell responses to a viral infection [47], and this finding corresponds to numerous other studies demonstrating an impact of probiotic supplementation, as recently reviewed [33,56,57]. BMDC from non-sensitized five-week-old offspring were differentiated over a 6 day period in GM-CSF as described [58], and are derived from existing progenitor cells within the developing hematopoietic compartment [59,60,61,62,63]. This approach allows for examination and analysis of their developmental and functional differences. The BMDC were then activated in vitro with respiratory syncytial virus (RSV; MOI = 1.0) or a TLR7 mimetic (imiquimod) for 24 h. BMDC from offspring exposed to prenatal Lj supplementation offspring had decreased the gene expression of numerous innate cytokines including, *Il6*, *Il12*, *Il1β* and *Tnfα*, all of which are associated with inflammatory responses after RSV infection and TLR7 agonist activation (Figure 4). Together, these studies suggest that maternal Lj supplementation leads to long-term alterations in BMDC grown from bone marrow progenitors, suggesting that BM programming is altered and may contribute to the subsequent responses to lung allergen challenges.

## 4. Discussion

Our findings support a model in which maternal probiotic supplementation is associated with effects that reshape offspring gut microbial biology and immune responses to environmental antigens and are associated with later-life susceptibility to inflammatory airway disease. In the present study, maternal Lj supplementation restricted to the mother prior to and during pregnancy was sufficient to: (i) attenuate offspring allergic airway pathology after cockroach allergen sensitization/challenge and associated pathology, (ii) alter offspring cecal microbiome that remained distinct at 5 weeks of age, and (iii) modify innate immune responsiveness in the offspring bone marrow compartment, evidenced by reduced inflammatory cytokine gene expression in BMDCs following RSV or TLR7 stimulation. Together, these outcomes argue that maternal Lj exposure does not merely mediate a transient gut microbiome change, but rather establishes a persistent biological effect that influences immune outcomes and disease penetrance well beyond the perinatal time frame.

At five weeks of age, prior to airway sensitization, offspring from Lj-supplemented dams exhibited clear separation in beta-diversity and a distinct phylum-level distribution characterized by shifts in Firmicutes and Proteobacteria and an enrichment of Verrucomicrobia (Akkermansia). The microbiome differences suggest that Lj exposure restructures broader anaerobic commensal communities, including taxa within Lachnospiraceae, Porphyromonadaceae, and the emergence or enrichment of Akkermansia, among others. This is consistent with the view that early-life perturbations and/or supplementation can shift microbiome development, establish different commensial communities, and alter the pool of microbial-derived metabolites encountered by the developing immune system [64,65,66]. The fact that Akkermansia, a mucus-associated microbe, was highly upregulated and associated with reduced allergen-induced disease, which is consistent with previous studies demonstrating that it is a beneficial microbe [67,68,69] and in itself a potentially important disease-modifying probiotic [70]. These conclusions are strengthened by and mechanistically aligned with recent work demonstrating that the maternal supplementation and the gut microbiome regulates offspring immunity to early-life RSV infection through coordinated effects on maternal and offspring microbiota and systemic metabolite profiles [47].

Functionally, PICRUSt2-based inference further supports the notion that maternal Lj supplementation establishes an altered metabolic environment due to the offspring microbiome. Although PICRUSt2 provides inferred functional capacity rather than measured metabolites, the Lj-associated expansion of anaerobic commensal communities (including Lachnospiraceae and Ruminococcaceae) together with predicted shifts in carbohydrate/glycan and amino-acid metabolic pathways suggests potential differences in microbiota-derived immunoregulatory metabolites. These include short-chain fatty acids (SCFAs), bile acid conversion, and tryptophan-metabolism that generates aryl hydrocarbon receptor ligands. Each of these metabolite classes can modulate epithelial barrier function and antigen-presenting cell cytokine programs, providing a plausible mechanistic link between maternal Lj-driven microbiome imprinting and reduced allergic airway pathology [71,72,73,74,75,76]. These data provide a strong rationale for future studies directly measuring key metabolites (e.g., SCFAs, bile acid pools, and tryptophan metabolites), especially in plasma, and linking them to defined immune endpoints that would limit the development of detrimental responses [77,78,79], such as modified inflammatory and immune modifying cytokines. Additionally, we and others have associated changes in serum metabolites with lung disease and altered pathophysiology, providing a link between early-life perturbations and long-lasting microbiome changes [47,80,81,82].

A major mechanistic insight from this work is that maternal Lj exposure is associated with the bone marrow-derived innate immune compartment. BMDC differentiated from supplemented offspring exhibited reduced induction of inflammatory cytokines in response to RSV or TLR7 stimulation relative to naive offspring, indicating that the offspring immune system is altered not only at mucosal sites but also at the level of progenitor-derived innate cell programming. This finding fits with emerging concepts of trained innate immunity, whereby early environmental exposures regulate transcriptional responsiveness via epigenetic and metabolic mechanisms [2,32,83,84,85]. In this context, maternal *Lj* may influence offspring through multiple, non-mutually exclusive pathways, including vertical transfer of microbes and altered early colonization dynamics, changes in maternal metabolites and microbial products reaching the fetus, altered maternal immune regulation that shapes fetal immune development, and/or altered microbe-derived metabolites encountered postnatally that feed back on hematopoietic development. Indeed, our early observations on maternal Lj supplementation indicated that the immune effects on offspring immunity in response to an early-life RSV infection were associated with the in utero exposure, as shown using cross-fostering modeling [47]. These concepts are also supported by studies linking early-life antibiotic use with susceptibility to asthma and associated lung disease later in life [4,86,87], as well as by environmental microbiome studies in rural vs. urban settings and in household dust studies [88,89,90,91,92].

From a translational perspective, these findings argue that maternal probiotic supplementation could represent a feasible strategy to reduce offspring risk of later allergic airway disease, particularly in settings where early-life immune dysregulation predisposes to asthma-like phenotypes. A key strength of the present study is that supplementation was limited to the dams, mirroring real-world feasibility where maternal interventions may be simpler and safer to implement than sustained infant dosing [93,94]. However, translation will require careful consideration of strain specificity, dosing, maternal diet, and baseline microbiome state, all of which can influence probiotic efficacy and downstream microbial ecology [95,96,97]. In addition, we did not assess differences in the inflammatory cell accumulation in these studies, which would also impact the response and could be a focus of future work. Regardless, these studies, along with many others using maternal supplementation, provide support that the relatively straightforward supplementation can have long-lasting beneficial outcomes on offspring.

An important limitation is the absence of independent litter-level replication in the microbiome and BMDC cohort. Because each maternal-treatment group was not consistently represented by multiple dams, maternal treatment was likely confounded with dam-specific influences. The observed differences therefore identify cohort-associated microbial and BMDC phenotypes but do not clearly establish that maternal Lj supplementation caused these differences. Confirmation in independently treated dams and litters will be required. Thus, these studies, while explorative, provide intriguing data for refining hypotheses and extending experimental questions. An additional limitation includes limited disease phenotyping for the allergic responsiveness, although the airway mucus, cytokine attenuation, and lung function assessments give a reasonable picture of altered responses.

In summary, our data support a model in which maternal Lj supplementation is consistent with a stable offspring gut microbiome that may reconfigure predicted microbial metabolic capacity and is associated with long-lasting changes in innate immune responsiveness centered. We speculate that the innate immune changes may include DC hematopoiesis [59,60,61,62,63], culminating in reduced susceptibility to allergic airway disease later in life. While the in vitro data with BMDC indicate that there is a general suppression of inflammatory cytokines, it is also possible that maternal Lj supplementation may provide an antigen-specific tolerogenic immune environment [98,99,100,101]. These findings reinforce the broader paradigm that maternal microbial exposures during pregnancy can shape offspring immune development and disease trajectories, and they provide a strong framework for mechanistic studies aimed at identifying the microbial and metabolic mediators that can be leveraged for preventive strategies against chronic allergic disease.

## 5. Conclusions

Maternal *Lactobacillus johnsonii* supplementation was associated with persistent changes in the offspring gut microbiome, reduced innate inflammatory responsiveness, and attenuated allergic airway disease. These findings support an intergenerational gut–bone marrow–lung axis through which maternal microbial exposure may shape later-life immune susceptibility. Further studies using independently treated dams and litters are required to establish causality and define the underlying microbial and hematopoietic mechanisms.

## Data Availability

The raw data supporting the conclusions of this article will be made available by the authors on request.
